# The Pre-Eclampsia Ontology: A Disease Ontology Representing the Domain Knowledge Specific to Pre-Eclampsia

**DOI:** 10.1371/journal.pone.0162828

**Published:** 2016-10-27

**Authors:** Satoshi Mizuno, Soichi Ogishima, Hidekazu Nishigori, Daniel G. Jamieson, Karin Verspoor, Hiroshi Tanaka, Nobuo Yaegashi, Jun Nakaya

**Affiliations:** 1 Department of Clinical Informatics, Tohoku University Graduate School of Medicine 2–1, Seiryo-machi, Aoba-ku, Sendai, Miyagi, Japan; 2 Department of Bioclinical Inforamtics, Tohoku Medical Megabank Organization, Tohoku University 2–1, Seiryo-machi, Aoba-ku, Sendai, Miyagi, Japan; 3 Department of Gynecology and Obstetrics, Tohoku University Graduate School of Medicine 1–1, Seiryo-machi, Aoba-ku, Sendai, Miyagi, Japan; 4 Biorelate Ltd., Manchester, United Kingdom; 5 Department of Computing and Information Systems, University of Melbourne, Parkville, VIC, Australia; Queen's University, CANADA

## Abstract

Pre-eclampsia (PE) is a clinical syndrome characterized by new-onset hypertension and proteinuria at ≥20 weeks of gestation, and is a leading cause of maternal and perinatal morbidity and mortality. Previous studies have gathered abundant data about PE such as risk factors and pathological findings. However, most of these data are not semantically structured. Clinical data on PE patients are often generated with semantic heterogeneity such as using disparate terminology to describe the same phenomena. In clinical studies, interoperability of heterogenic clinical data is required in various situations. In such a situation, it is necessary to develop an interoperable and standardized semantic framework to research the pathology of PE more comprehensively and to achieve interoperability of heterogenic clinical data of PE patients. In this study, we developed an ontology representing clinical features, treatments, genetic factors, environmental factors, and other aspects of the current knowledge in the domain of PE. We call this pre-eclampsia ontology “PEO”. To achieve interoperability with other ontologies, the core structure of PEO was compliant with the hierarchy of the Basic Formal Ontology (BFO). The PEO incorporates a wide range of key concepts and terms of PE from clinical and biomedical research in structuring the knowledge base that is specific to PE; therefore, PEO is expected to enhance PE-specific information retrieval and knowledge discovery in both clinical and biomedical research fields.

## Background

Pre-eclampsia (PE) is a clinical syndrome characterized by new-onset hypertension and proteinuria at ≥20 weeks of gestation [1, 2]. It affects 2–8% of all pregnancies, and is a leading cause of maternal and perinatal morbidity and mortality [3]. PE is a multi-systemic disorder targeting several organs, including the kidneys, liver and brain, and can cause anasarca, HELLP syndrome, cerebral edema, impaired liver function, abruption of placenta, intrauterine growth restriction, preterm delivery, maternal death and fetal death [1, 2, 4]. PE is believed to result from a complex interplay between genetic components and environmental factors. In previous studies, several risk factors have been reported for PE such as genetic variants of the angiotensin I converting enzyme [5], obesity [6] and use of antidepressants during pregnancy [7]. In terms of PE pathology, much knowledge has been accumulated such as failure of spiral artery remodeling, impaired extravillous trophoblast invasion [8], failure of maternal immune tolerance [8], placental damage by inflammatory stimuli [9], and dysfunction of maternal vascular endothelium [10]. However, the total image of PE pathology, such as the mechanism of genetic and environmental interactions, multisystem relationships and pathological changes according to progress of pregnancy, is still unclear. Because of its prevalence and gravity, a great deal of data about PE has accumulated in books, papers and databases, and the amount of data is steadily increasing. Some of these data, for instance a structured database of PE-related SNPs (PESNPdb [11]) are already well structured. However, most of these data are not structured with semantic annotation. On the other hand, clinical data of PE patients including diagnosis, treatments and observations are often generated using heterogeneous protocols, using disparate terminology to describe the same phenomena and using identical terms to describe disparate phenomena. For instance, the criteria for diagnosis and treatment for management of severe preeclampsia are not standardized across periods, countries and clinical facilities [2, 12–15].) In clinical studies, interoperability of heterogenic clinical data is required in various situations such as in the assembly of clinical data from multiple clinical facilities into one database system for multi-center clinical researches. Manual curation of heterogeneous data is a costly and non-scalable approach for studies involving thousands of patients. Therefore, reduction of semantic heterogeneity and achievement of interoperability are major informatics challenges [16, 17].

A knowledge-domain-specific ontology is a semantic framework which provides concepts and terminology in both biomedical and clinical researches. An ontology is a formal naming and definition of the concepts, terms, and interrelationships of entities that really or fundamentally exist for a particular knowledge domain [18]. In previous studies, ontologies have been developed for use as semantic frameworks in biomedical knowledge regions (e.g. Gene Ontology (GO) [19]), clinical knowledge regions (e.g. SNOMED-CT). Particular in clinical knowledge domain, Cross-disease ontologies such as Disease Ontology (DO) [20] and Online Mendelian Inheritance in Man (OMIM) [21] were previously developed and used to broad knowledge engineering tasks such as information retrieval, semantic annotation of data [22, 23], interoperability of databases [24], text classification of free written medical records [25] and structuring of unstructured data [26] while keeping its semantic identity [27].

Disease-specific ontologies were recently developed to create the structured space needed for more in-depth research on a disease, disease-specific ontologies, and a type of lower-level ontology, and to incorporate disease-specific concepts and terms; examples of these disease ontologies are the Alzheimer’s Disease Ontology (ADO) [28] and the Epilepsy and Seizure Ontology (EpSO) [29]. In previous studies, disease-specific ontology applied to text mining to modeling putative candidate pathological gene regulatory networks of Alzheimer’s disease, mining electronic medical record (EMR) to extract drug-usage and comorbidities of Multiple Sclerosis (MS) [30] patients and integrate clinical data of epilepsy patients from multiple EMRs to facilitate further investigation [31]. In these studies, shared terminology and a set of concepts in disease-specific ontologies plays a role as a semantic template for mapping terms from biomedical literature and clinical observations [32]. For example of EpSO, shared terminology of EpSO is used to reconcile differences in the terminology used for describing seizure events across EMRs. The EpSO also enables rendering of the correct signal data of electroencephalogram (EEG) segment with standardized event markings [33]. Same as both Alzheimer’s disease and epilepsy and seizure domain, like these biomedical and clinical informatics tasks would play important roles to facilitate PE researches; however no knowledge framework is currently capable to cover the complete domain of PE.

## Material and Methods

### Knowledge acquisition and conceptualization

Terms and concepts related to PE were collected from 40 articles. The criteria for literature selection were as follows: 1) include research articles, 2) published from Nov, 2013 to Aug, 2014 and 3) full text available via PubMed, 4) include epidemiological, genetic and clinical study 8) exclude review, case reports and nursing study. For knowledge enrichment, we include one study about animal model study and one cell model study. We select 41 articles from the pool of researches after applying literature selection criteria chronologically (S1 Table). Other 39 articles were about human research. Terms and concepts related to PE were collected from full texts including paper bodies and figure legends with a literature curation pipeline as follows.

### Literature curation pipeline

We use a literature curation pipeline to collect concepts and terms about specific knowledge domains from biomedical full text articles. All functions are performed by open source software. The pipeline collects gene names, gene variants, environmental factors and clinical features. The process by which this pipeline extracts information is as follows. 1) First, it extracts the full text from article PDF files with PDFx [34]. PDFx is a rule-based system designed to reconstruct the logical structure of scholarly articles in PDF form, regardless of their formatting style. 2) Next, it obtains genetic variants with TmVar [35] from an extracted full-text file. TmVar is a text-mining tool based on a conditional random field for extracting a wide range of sequence variants described at protein, DNA and RNA levels. 3) Then, it obtains gene names and protein names with BANNER [36]. BANNER is an open-source, executable survey of advances in biomedical named entity recognition. 4) Then, it obtains phenotypic features (e.g. disease states, complications and laboratory results) with text annotation to previous developed ontologies via NCBO BioPortal v4.0 REST service [37]. In this annotation process, we defined the terms annotated by least one of following four ontologies as phenotypic features; Human Phenotype Ontology (HP) [38], Disease Ontology (DO) [20], Online Mendelian Inheritance in Man (OMIM) [21] and Mammalian Phenotype Ontology (MP) [39]. 5) Finally, it obtains environmental factors with exact matching via the Environmental Factor Dictionary. The Environmental Factor Dictionary consists of 606 environmental factors such as high BMI and ethnicity. The factors of this dictionary were collected from 40 articles with manual curation until the increase in the number of factors ceased, or the factor base became saturated (S1 Fig).

### Construction of PE ontology

The PE ontology was constructed with concepts and terms collected through the literature curation pipeline. Whenever possible, we annotated terms to major biomedical and disease-specific ontologies and imported the annotated class from available ontologies using NCBO’s BioPortal. Available hierarchical structures of the concepts were also extracted along with the concepts themselves. The process of term annotation to other ontologies and import classes contribute to reduce semantic heterogeneity [40]. Ontologies used to import classes included the Alzheimer’s Disease Ontology (ADO), Medical Subject Headings (MESH), Systematized Nomenclature of Medicine—Clinical Terms (SNOMED-CT), Medical Dictionary for Regulatory Activities (MEDDRA), Online Mendelian Inheritance in Man (OMIM), Human Phenotype Ontology (HPO), Chemical Entities of Biological Interest Ontology (CHEBI), Health Level Seven Reference Implementation Model Version 3 (HL7)) and Exposure Ontology (ExO) [41]. We created preeclampsia knowledge domain specific classes using the terms have not matched to the classes of these ontologies. To ensure interoperability with existing and future biomedical ontologies, the PEO was based on Basic Formal Ontology (BFO) principles [42]. To describe disease-specific semantics, the PEO follows the class definitions and structures of root subdomains and axiomatic classes of the ADO.

The PE ontology was developed using the format of W3C standard Web Ontology Language (OWL2) (http://www.w3.org/TR/owl-guide/). The source code of the PE ontology is open and freely available under the Apache License 2.0.

### Reasoner run

To test with regard to formal consistency and absence of cycles in both individual objects and classes of objects, description logic reasoner (Fact++) was performed [43].

## Results

### Overview of PEO

**Fig 1:**
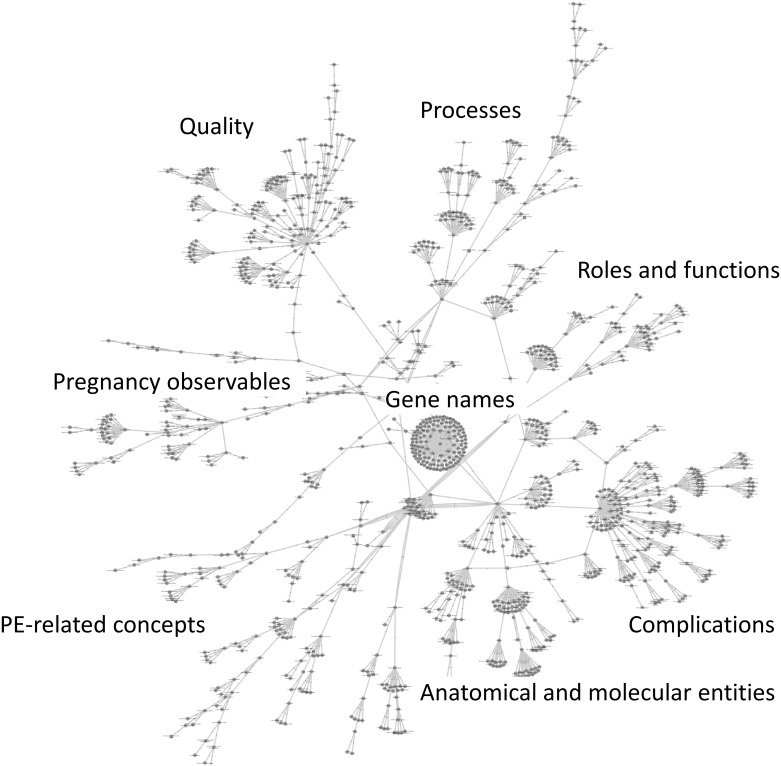
Overview of the PEO. Black dots represent a class and silver lines represent relationships between two classes. PEO consist of 1,251 classes and 1,507 relations.

**Fig 2:**
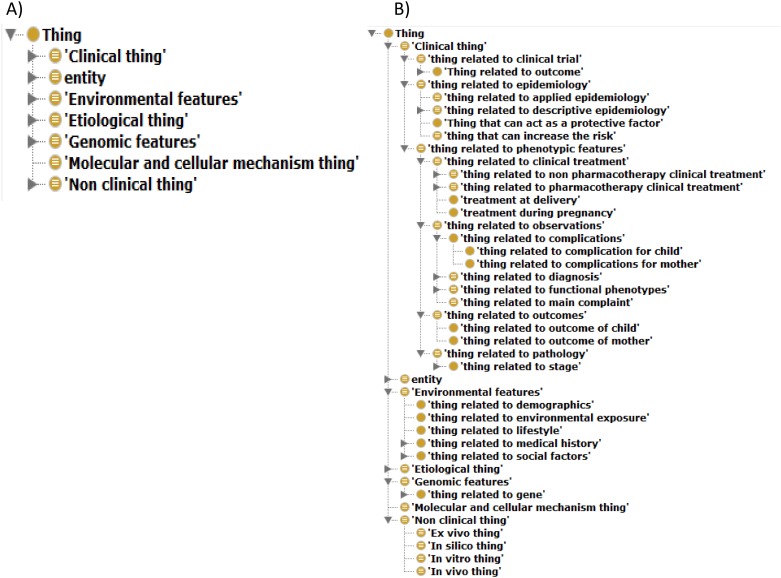
Hierarchy of concepts in PEO. (A) Root concepts in PEO. Root concepts consist of “Clinical”, “Nonclinical”, “Etiological”, “Molecular and cellular mechanism”, “Genomic features” and “Environmental features”. (B) Extracted concepts tree in PEO. PE-specific concepts such as “Thing related to complication of child” and “Thing related to complication of mother” were implemented.

The developed PEO consists of 1,251 classes and 1,507 relations. Overview of the entire ontology is shown in Fig 1 as network style graph. As described in Fig 1, broad ranges of terminology are covered by the PEO. The aspect of terminology is described in “The range of terminology covered by the PEO” section. On the structural features of PEO, the root concepts of the PEO include “Clinical”, “Nonclinical”, “Etiological”, “Molecular and cellular mechanism”, “Genomic features”, “Environmental features” and “entity”. These root concepts are shown in Fig 2A. We summarize number of classes under the root classes and their maximum depth in Table 1. To ensure interoperability with other ontologies, the core structure of PEO was compliant with the hierarchy of BFO. In this context, formal concepts and hierarchies such as bfo:continuant and bfo:occurrent were imported from BFO. The PEO covers a wide range of key concepts and terms of PE specific to both clinical and biomedical research to structure the knowledge domain specific to PE. The latest version of the PE ontology is available for visualization and downloading from NCBO’s BioPortal: (http://bioportal.bioontology.org/ontologies/PE-O). Details of the ranges of data covered and hierarchical structures of concepts and terms are described below.

**Table 1:** Summary of the number of classes under the root classes and their maximum depth in the PEO.

Root concept	Num. of lower classes	Max depth of lower class
Clinical thing	48	6
Environmental features	13	2
Etiological thing	1	1
Genomic features	6	4
Molecular and cellular mechanism thing	0	0
Non clinical thing	4	1
Entity	1,087	12

### Structure and contents

To cover a wide range of biomedical concepts, “Clinical thing”,”Nonclinical thing”, “Etiological thing”, “Molecular and cellular mechanism thing” and “entity” were imported from ADO as general concepts of disease-specific ontologies. To cover PE specific concepts, “Genomic features” and “Environmental features” were newly created. Subclasses under “Clinical thing” include concepts about phenotypic features, epidemiology and clinical trials. Most of the phenotypic features were newly created, and included the concepts pathology, outcomes, treatment options, clinical observations and clinical outcomes. Some of these classes have subclasses related to PE and gynecology such as “treatment during pregnancy”, “outcome of child” and “outcome of mother”. Concepts and hierarches under “Nonclinical thing” and “Etiological thing” were fully compliant with ADO. “Genomic features” cover concepts about genes, transcripts and genomic variants to describe genomic risk factors and pathological genomic features related to PE. “Environmental features” included concepts about demographics, medical history, environmental exposure, lifestyle and social factors identified as risks (e.g., “thing related to social factors”.) Extracted views of subclasses under root concepts are shown in Fig 2B. The semantic relationships “is a,” “has a,” and “part of” were used to define relation types between pairs of concepts.

### The range of terminology covered by the PEO

**Fig 3:**
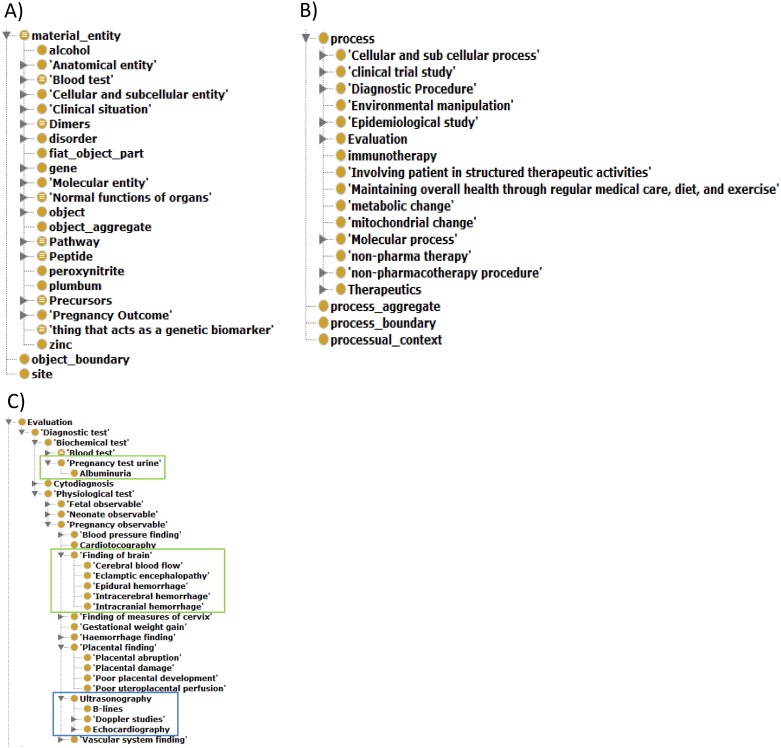
Hierarchy of terminology in PEO. (A) Hierarchy of terminological super classes about objects. (B) Hierarchy of terminological super classes about processes. (C) Examples of PE-specific terminology. Terms in blue box are gynecology-specific such as “Ultrasonography”. Terms in green box are PE-specific such as “Pregnancy test urine” and “Finding of brain”.

Over 1,000 sub-classes of “entity” cover a wide range of terminology related to PE such as clinical and pathological entities. To interoperability with other biomedical ontology, top level-classes and sub-classes of entity ‘inherited’ the top-level concepts of the basic format ontology (BFO). The top-level concepts consist of bfo:continuant and bfo:occurrent. Sub-classes of bfo:continuant consist of non-processual classes. For example, contexts such as the clinical context (e.g., “clinical main complaint context”) are modeled as subclasses of bfo:DependentContinuant. The major part of sub-classes of bfo:DependentContinuant are arranged under bfo:quality. bfo:quality consist of terminology about “being cannot exist without something” such as innate character (e.g., ethnicity) and status (e.g., education). Other terminologies including pathological genes (e.g., “CXCL2”), anatomical entities (e.g., “Chorionic villi structure”), pregnancy outcomes (e.g., “Miscarriage”) and complications (e.g., “Intrauterine growth retardation (IUGR)”) are modeled as subclasses of bfo: IndependentContinuant. bfo: IndependentContinuant consists of realizable entities. To normalize, gene names are mapped to appropriate entry of Entrez gene. Almost of all other terminology are refer entry of major biomedical ontologies such as SNOMED-CT and MEDDRA. Sub-classes of bfo:occurren” consist of processual and spatial-temporal terminology such as diagnostic procedure (e.g., “Serum total HCG measurement” and “diagnostic procedures such as ultrasonography”), molecular processes (e.g., “Oxidative stress”), temporal (e.g., Pregnancy time period). The details of terminological hierarches are shown in Fig 3A and 3B. An example of PE-specific terminology is shown in Fig 3C. In the process of terminology for equipment, synonyms of terms were enriched to one class using both manual and automated methods. For this purpose, services provided by the National Center for Biomedical Ontology (http://bioportal.bioontology.org/) were used to enrich synonym information. Reference ontologies were limited to noteworthy biomedical ontologies described in the “Construction of PE ontology section of Materials and Methods.

### The proportion of the PE knowledge domain specific classes versus the other

In the PEO, PE-related concepts and terms were structured in a PE-specific semantic hierarchy through class import and synonym enrichment via NCBO BioPortal v4.0 REST service. Other parts of the PEO were newly created through literature curation, by which 411 classes (32.8%) were newly created.

### PEO review and feedback

Evaluation of the PEO was performed by an obstetrician. The reviewer pointed out insufficiency the terminology about ultrasonography and Doppler. According to the reviewer’s comment, we extracted important terminology such as “Pulsatility index” using William’s obstetrics 24th edition, which is standard textbook of obstetrics worldwide.

## Discussion

### Features of the PEO

In this research, we developed the first draft of PE ontology (PEO) from PE-related papers used as resources for concepts and terms. The PEO was designed to provide maximum coverage of PE. To ensure interoperability with other biomedical ontologies, the core structure of the PEO was compliant with the basic formal ontology (BFO) hierarchy.

### PE specific classes of PEO

The PEO has 840 imported classes from other biomedical ontologies and 411 newly created ontology. These newly created classes represent preeclampsia knowledge domain specific terminology such as “Fetal thrombotic vasculopathy”, “Hypercoiled umbilical cord” and “Decidual Arteriopathy”. This terminology will contribute to future knowledge engineering tasks such as comorbidity extraction and query reasoning to extract PE-specific dataset from patient’s electronic medical records (EMRs).

### The terminology for comorbidity and PE-related genes

About 200 terms of complications such as “HELLP syndrome” and “Hyperlipidemia” are accumulated in PEO. These terms of complications refer major biomedical ontology such as SNOMED-CT and MEDDRA. The terminology for complications of PE contributes to comorbidity analysis tasks such as capture comorbidities of patients from EMR. In the PEO, 123 gene names are accumulated. The terminology about PE-related genes would contribute biomedical tasks such as gene set enrichment analysis.

### Compare to other disease-specific ontologies

In the previous works to build Alzheimer’s disease ontology (ADO)), automatically processing was adapted to process over 50,000 abstracts [28]. The ADO consists of 1,565 classes and 2,401 relationships. In this study, we adapted manual curation from 40 full-texts. The PEO consist of 1,251 classes and 1,507 relationships. This result indicates that 40 full-texts are not too few to disease-specific ontology. Both manual curation and automatically processing have both good and bad points. Manual curation is accurate but costly and non-scalable approach. Automatically processing is cost-effective but has possibility of contamination of unnecessary terms.

### The scenario of applications

The PEO covers a broad range of knowledge derived from clinical research (e.g. diagnosis and treatment) and biomedical studies (e.g. molecular aspects and pathways); therefore, applications for knowledge engineering of both the clinical and biomedical knowledge domains can be expected. The scenario in clinical research is similar to those of previously developed disease-specific ontologies such as those for epilepsy and seizures [44]. In this scenario, PEO works as a knowledge-domain-specific semantic framework under a large ontology that describes all disease areas [20]. As a specific example, PEO could help to make connections among PE-specific data such as medical history, changes in blood pressure during pregnancy and findings of fetal echo in electronic health record systems. On the other hand, the scenario in biomedical research is slightly different between previous biomedical ontologies. In this scenario, PEO would work as an accelerator of PE-specific big data to knowledge which includes PE-specific multi organ dysfunction and relationships among multi-factorial genetic-environmental factors in both biomedical and clinical informatics. Such works which make connections among phenotype, genome and environmental factors are major challenge in the biomedical research area. [45]. In addition, PEO will be used in data mining and knowledge discovery tasks over large-scale scientific texts and datasets in both clinical and biomedical research areas.

## Conclusions

A Preeclampsia Ontology (PEO) was developed from concepts and terms of PE-related literature. This PEO is the first semantic framework to cover in detail the various aspects of PE-related clinical and biomedical research knowledge domains. The PEO consists of 1,251 classes representing both clinical and biomedical concepts and terms, and is expected to be used for PE-specific data mining and knowledge discovery in both clinical and biomedical research.

## Supporting Information

S1 Fig The increment of environmental factors according to the number of papers used in our environmental factor dictionary. (PPTX)

S1 Table The full list of 41 papers to collect terms and concepts related to PE for the development of PEO. (XLSX)
