# Supplementary material for: The Pre-Eclampsia Ontology: A Disease Ontology Representing the Domain Knowledge Specific to Pre-Eclampsia
Source: PLoS One. 2016 Oct 27;11(10):e0162828. doi: 10.1371/journal.pone.0162828 (PMC5082890; doi:10.1371/journal.pone.0162828)
Supplement: S1 Fig — (PPTX) [file pone.0162828.s001.pptx]

## Slide 1
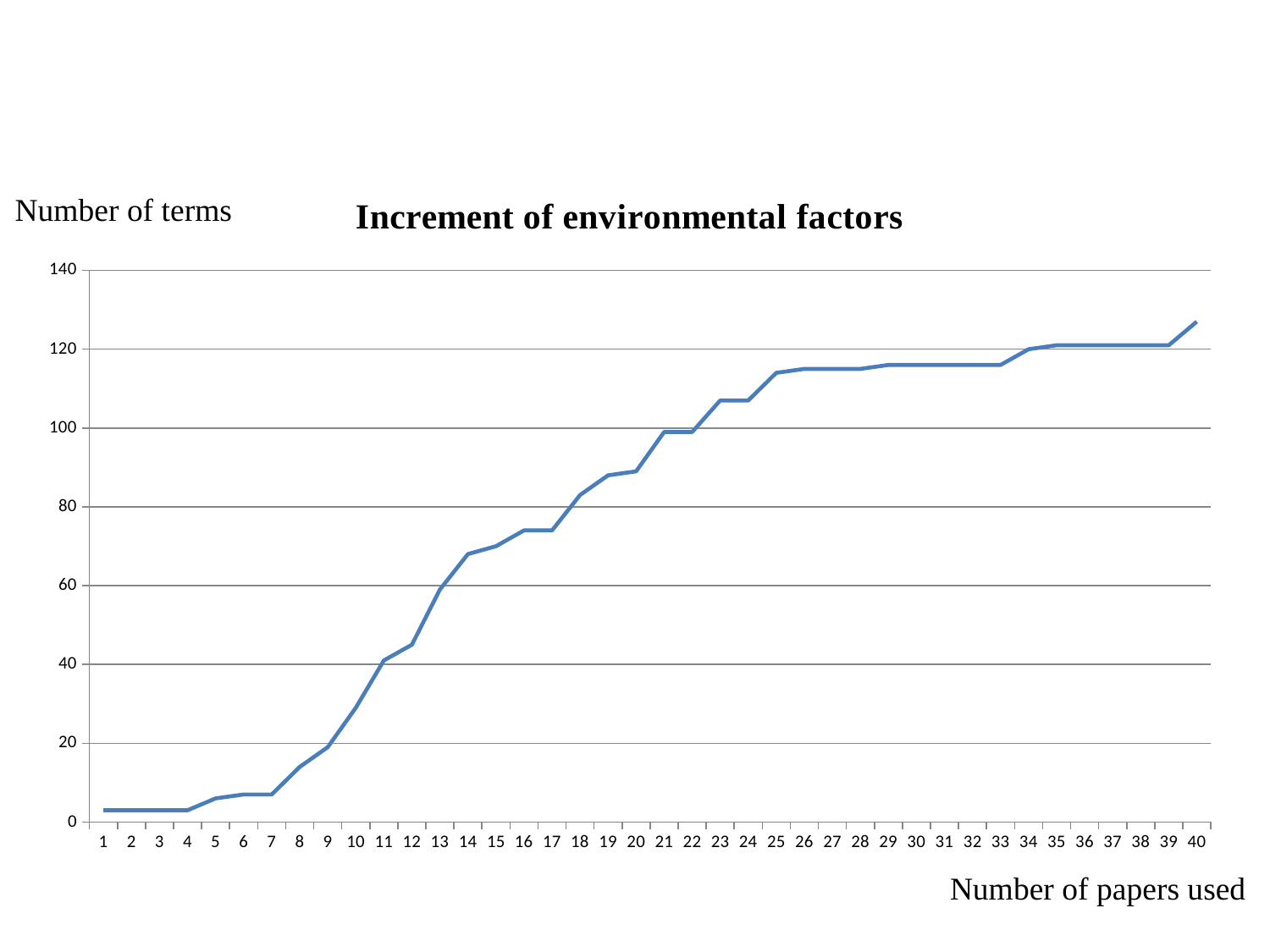

### Chart: Increment of environmental factors
| Category | Increment of environmental factors |
|---|---|Number of terms
Number of papers used
